# Ethanol Production from Oil Palm Trunk: A Combined Strategy Using an Effective Pretreatment and Simultaneous Saccharification and Cofermentation

**DOI:** 10.1155/2021/2509443

**Published:** 2021-12-23

**Authors:** Agustin Krisna Wardani, Aji Sutrisno, Titik Nur Faida, Retno Dwi Yustina, Untung Murdiyatmo

**Affiliations:** ^1^Department of Agricultural Product Technology, Universitas Brawijaya, Malang 65314, East Java, Indonesia; ^2^Department of Research and Development, PT Sampoerna Agro Tbk, Palembang 30128, South Sumatera, Indonesia; ^3^Indonesian Ethanol Association, Malang, Indonesia

## Abstract

**Background:**

Oil palm trunk (OPT) with highly cellulose content is a valuable bioresource for bioethanol production. To produce ethanol from biomass, pretreatment is an essential step in the conversion of lignocellulosic biomass to fermentable sugars such as glucose and xylose. Several pretreatment methods have been developed to overcome biomass recalcitrance. In this study, the effects of different pretreatment methods such as alkali pretreatment, microwave-alkali, and alkaline peroxide combined with autoclave on the lignocellulosic biomass structure were investigated. Moreover, ethanol production from the treated biomass was performed by simultaneous saccharification and cofermentation (SSCF) under different temperatures, fermentation times, and cell ratios of *Saccharomyces cerevisiae* NCYC 479 and pentose-utilizing yeast, *Pichia stipitis* NCYC 1541.

**Results:**

Pretreatment resulted in a significant lignin removal up to 83.26% and cellulose released up to 80.74% in treated OPT by alkaline peroxide combined with autoclave method. Enzymatic hydrolysis of treated OPT resulted in an increase in fermentable sugar up to 93.22%. Optimization of SSCF by response surface method showed that the coculture could work together to produce maximum ethanol (1.89%) and fermentation efficiency (66.14%) under the optimized condition.

**Conclusion:**

Pretreatment by alkaline peroxide combined with autoclave method and SSCF process could be expected as a promising system for ethanol production from oil palm trunk and various lignocellulosic biomass.

## 1. Introduction

The development of biorefining process technologies to produce biofuels from renewable biomass sources represent a key tool to perform the transition from a fossil fuel-based economy to a novel bioeconomy that looks for a more efficient and sustainable global development [1, 2]. According to the biorefinery concepts, there are many kinds of biomass feedstocks used, such as sugar, starch, aquatic biomass, organic residues, and lignocellulose, or oil-containing crops, can be converted into bioenergy products. Bioethanol is one of renewable biomass energy produced via sugar fermentation and can be a potential source of sustainable fuel. It has been proposed that an alternative feedstock for biofuel is wasted crops, replacing the traditional starch crop and can avoid conflicts with human food uses.

Indonesia is one of the largest producers of palm oil in the world. In 2010, oil palm plantations produced 22 million tonnes of Crude Palm Oil (CPO), while in 2011, it was 23.5 million tonnes [3]. By 2020, Indonesia plans to double the current production of CPO to 40 million tonnes annually and expand its oil palm plantation portfolio by additional 4 million hectares. Along with the growing palm oil industry, it creates the availability of palm oil residue, including oil palm trunk (OPT) waste, which is considered a great potential source of renewable energy. Oil palm trunk contains approximately 70% sugar and 30% (w/w) cellulosic residue. This indicates that OPT is a very promising material to be used as feedstock for second-generation ethanol production.

In general, the production of ethanol from lignocellulose requires several stages, including delignification, saccharification to liberate fermentable hexoses and pentoses of polysaccharides, released sugar fermentation, and distillation stage to separate the ethanol [4, 5]. Delignification is the essential step to effectively prepare cellulose to be used by fermenting microorganism for ethanol production [6, 7]. Some strategies for pretreatment such as microwave, ultrasound, deep eutectic solvent, irradiation, and ionic liquids methods have been applied to decrease the recalcitrance of biomass [8–10]. However, the major problem is the involvement of high capital cost and the low effectiveness for lignin removal. In this study, some pretreatment methods, such as alkali, alkali-microwave, and alkaline peroxide combined with autoclaves, were used to remove the lignin content of the oil palm trunk. In addition, the simultaneous saccharification and cofermentation (SSCF) system was chosen to maximize the utilization of hexose and pentose sugars by *Saccharomyces cerevisiae* NCYC 479 and *Pichia stipitis* NCYC 1541. The SSCF system has some additional advantages as follows: (1) the existence of yeast and enzyme complexes together reduce the accumulation of glucose and short cellulose oligomers which can inhibit the enzyme and thus be able to increase the yield of ethanol and saccharification rate; (2) the use of one bioreactor can reduce investment costs; (3) the presence of ethanol in the bioreactor and the rapid consumption of sugar by yeast reduce the risk of contamination [11–13]. Simultaneous saccharification and cofermentation (SSCF) system is influenced by many factors, such as cell ratio, temperature, and fermentation time [14]. In the current study, optimization of fermentation process parameters for *S. cerevisiae* and *P. stipitis* coculture was performed using RSM. Parameters examined in this study were cell ratio between *S. cerevisiae* and *P. stipitis*, culture temperature, and fermentation time. Cell ratio between *S. cerevisiae* and *P. stipitis* related to assimilation rate of glucose and xylose by the yeasts. Thus, this parameter is considered a major factor in ethanol production efficiency. Temperature and fermentation time is also a key factor in yeast growth. Therefore, these parameters were also optimized for ethanol fermentation [15, 16]. Response Surface Methodology (RSM) with a full factorial central composite design (CCD) was applied to optimize fermentation to maximize ethanol production and fermentation efficiency. This method intends to find an appropriate function to predict the response (ethanol content and fermentation efficiency) and determine the value of the independent variables (cell ratio, temperature, and fermentation time) that provide an optimal response.

## 2. Materials and Methods

### 2.1. Materials

The oil palm trunk (OPT), estimated to be 25 years old, was collected from the plantation of PT Sampoerna Agro Tbk., Indonesia. Ten cm in thickness of disks trunk was taken from the middle part of each trunk, which ranged from 10 to 12 m in length. A laboratory-scale crusher was used to make disks trunk into small particles. The sap was squeezed from the disks using a laboratory-scale press at 250 Bar. The free sugars remaining in the disks were removed by twice washing with distilled water. The moisture content of crushed disks was reduced to <5% using an oven (60°C for 48 h). The dried disks were pounded manually into small pieces until the mixed fiber was ready to separate into parenchyma (PA) and vascular bundle (VB) by using 30 mesh (0.6 mm) and 80 mesh screen (0.2 mm). The retained particles on the 80 mesh screen (VB) were used as the raw material for ethanol production.

### 2.2. Alkali Pretreatment

Ten percent of raw materials (dried VB) were pretreated with dilute sodium hydroxide (NaOH) at a concentration of 5% (w/v) NaOH. Then, the mixtures were heated at 150°C for 3 h. The mixture was filtered to separate solid residues and thoroughly washed with distilled water to neutral pH. Finally, it was dried in the oven at 105°C for 48 h.

### 2.3. Alkali-Peroxide Pretreatment

Raw materials were pretreated with 250 ml of H_2_O_2_ 5% (v/v) solution in an autoclavable bottle. The mixture was adjusted to pH 11.5 using sodium hydroxide (NaOH) and incubated at room temperature for 3 days. To remove the moisture, it was heated at 121°C for 15 minutes, 1 atm, using autoclave. After heating, the raw material slurry was filtered to recover the insoluble solids. The solids were washed with distilled water until the pH of the solid became neutral. The washed treated raw materials were dried in a drying oven at 105°C for 48 h. After drying, the moisture content of treated raw materials was measured.

### 2.4. Alkali-Microwave Pretreatment

Raw materials (dried VB) were pretreated with dilute sodium hydroxide (NaOH) at a concentration of 5% (w/v) NaOH and at solid loading of 10%. Then, the mixtures were placed in an open 250 ml glass beaker and exposed to microwave radiation at 400 watts for 30 minutes and 800 watts for 80 minutes. The mixture was filtered to separate solid residues out. The solid residues were thoroughly washed with distilled water to neutral pH and dried in the oven at 105°C for 48 h.

### 2.5. Chemical Analysis Methods

The moisture content of raw material was determined by drying at 105°C for 48 h. The chemical composition of oven-dried untreated and pretreated raw material was analyzed following the National Renewable Energy Laboratory (NREL) Chemical Analysis and Testing Standard Procedure. The cellulose and hemicellulose content were determined by the methods of Van Soest et al. [17]. The lignin and starch content were analyzed according to Sluiter et al. [18] and Sluiter and Sluiter [19] in NREL Chemical Analysis and Testing Standard Procedure. The ethanol content was determined using an ethanol assay kit under standard conditions according to the manufacturer's instruction (Megazyme, K-ETOH 01/I4, Ireland). Monosaccharide components were quantified by high-performance liquid chromatography (HPLC; Shimadzu corp. Kyoto, Japan), with a refractive index detector (Shimadzu RID-10A) on a CLC-NH_2_(M) 25 cm operated at room temperature. These analytical values are shown as the means of duplicate experiments. Scanning electron microscopy (SEM) (FEI inspect-500) was employed to investigate the morphological properties untreated and treated raw material. The specimen for SEM was prepared by Au-Pd coating.

### 2.6. Enzymatic Hydrolysis

The treated raw material was hydrolyzed using a commercial complex cellulase (Cellic Ctec2, Novozyme, Denmark) and supplemented with complex hemicellulase Cellic Htec2 (Novozyme, Denmark). Cellic Ctec2 activity was estimated as 168,18 FPU/ml enzyme per gram substrate according to the filter paper assay [20] using National Renewable Energy Laboratory (NREL) Chemical Analysis and Testing Standard Procedure. The xylanase activity of Cellic Htec2 was estimated as 110 units/ml according to the assay method of Bailey [21]. Enzymatic digestibility of treated raw material was performed at 50°C with shaking 120 rpm in a 250 ml flask containing 5% (w/v) treated raw material, in the presence of 50 mM sodium acetate buffer (pH 5) with a working volume of 10 ml. The reaction was initiated by mixing 31.65 FPU/ml of Cellic Ctec2 and 1.6 units/ml of Cellic Htec2 per gram substrate. The released reducing sugar concentration was analyzed based on the amount of liberated reducing sugars using 3,5-dinitrosalicylic acid (DNS) method [22].

### 2.7. Microorganism and Inoculum Preparation

Hexose-utilizing yeast, *Saccharomyces cerevisiae* NCYC 479, and pentose-utilizing yeast, *Pichia stipitis* NCYC 1541, were obtained from National Collection of Yeast Culture (NCYC), Norwich, UK. The culture was maintained at 4°C on a yeast peptone dextrose agar consisting of yeast extract, 10 g l^−1^; peptone 20 g l^−1^; glucose 20 g l^−1^, and agar, 15 g l^−1^ at pH 5.0. Cells were grown in 150 ml Erlenmeyer flask containing 50 ml of YPD medium (1% yeast extract, 2% peptone, and 2% glucose) in a shaker incubator at 30°C with 100 rpm. Following 20 h growth, the broth was centrifuged and inoculum was prepared corresponding to 1.0 g l^−1 ^cells.

### 2.8. Optimization of Ethanol Fermentation

The experimental design and statistical analysis of fermentation were performed according to the RSM using Design-Expert software Version 7.1.5, Stat-Ease, Minneapolis, 2008. Central Composite Design (CCD) [23] was employed to study the combined effect of three independent variables: temperature (*X*_1_), fermentation time (*X*_2_), and cell ratio of *S. cerevisiae* and *P. stipitis* (*X*_3_). RSM experimental design for ethanol fermentation parameters optimization contains a lower and higher level of variables, that is, temperature (25, 30, and 35°C), fermentation time (3, 5, and 7 days), and cell ratio of *S. cerevisiae* and *P. stipitis* (0.25 : 0.75, 0.50 : 0.50, 0.75 : 0.25). The dependent variable selected for this study was ethanol concentration (*Y*_1_) and efficiency fermentation (*Y*_2_). In the CCD, the total number of experimental combinations was 2^*K*^ + 2*K* + *n*_0_, where *K* is the number of independent variables and *n*_0_ is the number of repetitions of the experiments at the center point, which indicated that 20 experiments were required for this procedure. The CCD contains a total of 20 experiments with five-level full factorial design and replications of the central points and axial points (Table 1). The divergences for each factor assessed were split into linear, quadratic, and interactive components and represented using the second-order polynomial function:

(1) $$\begin{array}{c} Y=b_{0}+b_{1}X_{1}+b_{2}X_{2}+b_{3}X_{3}+b_{12}X_{1}X_{2}+b_{13}X_{1}X_{3}+b_{23}X_{2}X_{3}+b_{11}X_{12}+b_{22}X_{22}+b_{33}X_{32}, \end{array}$$

where *Y* is the predicted response variable; *X*_1_, *X*_2_, *X*_3_ are independent variables; *b*_0_ is the offset term; *b*_1_*, b*_2_*, b*_3_ are linear effects; *b*_12_*, b*_13_*, b*_23_ are interaction terms; and *b*_11_*, b*_22_, *b*_33_ are squared effects. The significance of all terms in the polynomial functions was assessed statistically using F-value at a probability (*P*) of 0.05. The regression coefficients were then used to generate contour maps from the regression models. Validation was performed after the optimum fermentation condition for ethanol fermentation was obtained.

## 3. Results and Discussion

### 3.1. Separation of Vascular Bundle and Parenchyma

Separation of the vascular bundle (VB) and parenchyma (PA) of oil palm trunks was performed by cutting, size reduction, pressing, drying, and sieving. According to Darwis et al. [24], the vascular bundle has a higher density in the central part than the bottom of oil palm trunks. The chemical composition of palm trunks after 30 mesh (595 *µ*m) sieving is presented in Table 2.

Further sieving by using 80 mesh (177 *µ*m) was performed to separate starch from VB. Noor et al. [25] stated that the starch palm trunks size approximately 14.5 *µ*m. By further sieving (80 mesh), it is expected that starch will be eliminated. The chemical composition after 80 mesh (177 *µ*m) sieving is presented in Table 3.

It was found that the level of lignin and cellulose was higher in ≥177 *µ*m particles than in particles which have sizes less than 177 *µ*m. Thus, it is assumed that particle that did not pass in 80 mesh sieving is VB with high cellulose content. However, it still contained starch about 20.19%, which may decrease the effectiveness of delignification. Delignification in this study was done chemically and physically (heating). The effectiveness of delignification will decrease due to starch gelatinization, which occurs during heat treatment. The gelatinization temperature of palm starch is 52.4–72.1°C [25]. To solve this problem, enzymatic hydrolysis using *α*-amylase is selected to be applied to decrease starch content in the preparation of raw material prior to delignification [26, 27].  Table 4 shows the chemical composition of raw material after enzymatic hydrolysis.

Hydrolysis treatment led to a reduction in lignin and starch while an increase in cellulose and hemicellulose. Starch has been reduced to 29.47%, while cellulose and hemicelluloses have been increased until 8.51% and 5.28%, respectively.

### 3.2. Delignification

There are three methods of delignification, which were applied to the lignocellulosic palm trunks, which were alkali and heat treatment (NaOH 5% w/v, oven 150°C for 3 hours); alkali-microwave (NaOH 5% w/v, 400 watts for 30 min); and alkaline peroxide and heat treatment (NaOH 5% w/v, 121°C for 15 minutes). Alkaline conditions were chosen because of their advantages which include the following: (1) impact on the degradation of sugars smaller when compared to the delignification in acidic conditions, (2) ability to dissolve the lignin greater than in acidic conditions, and (3) a small effect on the crystallinity of cellulose [28, 29]. The comparison of lignin content before and after delignification is presented in Table 5.

**Figure 1:**
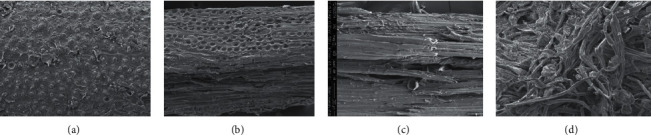
SEM images of untreated raw materials (a); pretreated raw materials with NaOH 5%, 150°C for 3 hour (b); pretreated raw materials with microwave-alkali (NaOH 5% w/v, 121°C for 15 minutes) (c); pretreated raw materials with H_2_O_2_ 5%; pH 11.5; 121°C for 15 min (d).

The result shows that the combination of alkali and heat treatment did not cause a significant lignin reduction, as well as in the alkali treatment with microwave. A significant lignin reduction (80.88%) was found in alkaline peroxide and heat treatment. By this pretreatment, the complex matrix is destroyed, and the enzymes' access to the carbohydrates is facilitated. Senila et al. [30] reported that the solid yields for the used autohydrolysis pretreatments were found to be between 62.2 and 71.0% (165°C) and 52.3 and 64.3% (180°C), respectively.  Figure 1 shows the physical change of raw materials before and after delignification.

Figure 1 provides an insight into the variation structure change affected by some methods of pretreatment. Alkali treatment with a hot oven causes physical changes in the surface (Figures 1(a) and 1(b)). The lignocellulose surface of the bulge-shaped section contains an accumulation of lignin [31].  Figure 1(b) shows that lignin has been degraded in the lignocellulosic surface. Gould et al. [31] and Kumar and Sharma [32] also explained that lignin is a compound that glue the vessels and restrict file microfibril cellulose in plant cell walls. A thin layer between cellulose microfibrils also is a complex of lignin and hemicellulose.  Figure 1(c) shows that microwaves-alkali treatment led to the degradation heat penetration because the interaction of microwaves with water molecules is higher than the heat penetration of a hot oven. Physical changes of raw materials after delignification with alkaline peroxide and heat treatment (1D) differ significantly from the condition of raw materials before treatment (Figure 1(a)).  Figure 1(d) shows that alkaline peroxide and heat treatment caused the degradation of lignin in the surface as well as between the cellulose microfibrils more leverage than the treatment of alkali with a hot oven or by microwaves [4, 33]. It is proven that cellulose microfibrils have successfully broken down. Singh et al. [29] also explained that the removal of lignin causes cell separation vessel beam, thus forming the cellular structure are linked to each other long in the longitudinal direction. Lignin decomposition may occur due to rupture *α*-aryl ether bond of monomer polyphenols constituent lignin. Therefore, treatment with hot alkaline peroxide delignification was chosen as the appropriate method to be applied to lignocellulose palm trunks. The advantages of alkaline peroxide delignification method with heat treatment do not require high pressure and expensive equipment and can significantly degrade lignin [34]. Changes in the chemical composition of raw materials after delignification with alkaline peroxide and heat treatment (121°C, 1 atm, 15 minutes) are presented in Table 6.

Table 6 explains that alkaline peroxide decreased lignin content to 3.39% and reduced hemicellulose content to 10.41%. In addition, alkaline peroxide increased cellulose content to 72.42%, suggesting that cellulose is readily hydrolyzed into sugars. Singh et al. [29] stated that the acetyl group is a side chain of the main structure of xylan. The removal of acetyl groups from the raw materials can improve the accessibility of the enzyme to cellulose and xylan.  Table 7 shows the effect of alkaline peroxide and heat treatment on the digestibility of substrate.

Table 7 shows that the content of reducing glucose in raw materials increased significantly (93.22%) after delignification. Lignin reduction was also observed to 3.39%. It was confirmed that the lignin reduction was a success and led to increasing the accessibility of cellulase to the substrate. Another study showed that alkaline pretreatment was conducted on sugarcane bagasse, reducing lignin content by 7.16% [35]. Furthermore, Aguirre-Fierro et al. [36] applied a high-pressure CO_2_–H_2_O mixture at various temperatures yielding 75.8 mol % of the polysaccharides present in bagasse. The combined effect of steam exploded, and acid hydrolysis was performed to obtain the high reducing sugar (77 g/L) [37].

### 3.3. Optimization of Ethanol Production Efficiency

The results presented in Table 1 revealed the 20 combinations along with their responses in terms of final ethanol production and fermentation efficiency. Among the 20 RSM combinations, maximum ethanol production was achieved in the combination of fermentation condition at 30°C, 5 days incubation with the same cell ratio at 0.5:0.5 (S. cerevisiae and P. stipitis). Saccharification and cofermentation simultaneously were performed preceded by prehydrolysis, which was conducted at 50°C for 8 hours. Prehydrolysis intended for fermentation can be initiated on the condition of the substrate and the yeast mix well [38]. Prehydrolysis processes were evaluated based on the changes of the physical substrate and sugar content. Inoculation of yeast into the substrate was performed after the substrate achieved a sugar content that can be used to initiate the fermentation. Cell ratio of *S. cerevisiae/P. stipitis*, culture temperature, and fermentation time were analyzed to determine its relationship to the response prior to the optimization stage [39]. The optimization stage was done after the independent and dependent variables proved to have a quadratic relationship [40]. Optimization of saccharification and cofermentation was done based on the central composite design response surface method. Data experimental results were analyzed using the Design-Expert program to predict regression (statistical model) response data. The independent variables, that is, cell ratio, temperature, and fermentation time, were optimized, whereas the observed response is ethanol and fermentation efficiency. The efficiency of fermentation is an additional response, which is the development of content data of ethanol. Fermentation efficiency value was calculated based on the concentration of ethanol divided by glucose levels change until the end of fermentation (Δ substrate) and multiplied by 100%. Optimization experiment design and ethanol production performance of *S. cerevisiae* and *P. stipitis* coculture are presented in Table 1.

Statistical model in Software Design Expert consists of a quadratic model, linear, 2FI (interaction of two factors), and cubic [41]. Selection of the most appropriate statistical model to determine the optimum response is based on the evaluation order of the sum of squares (sequential model of sum squares), inaccuracies testing model (lack of fit test), and summary statistics (model statistical summary) [40].  Table 8 provides the profile of xylose and glucose of *S. cerevisiae/P. stipitis* coculture fermentation.

The data were put in equation (1), and the resulting regression second polynomial equations with significant factors for two responses (equations (2) and (3)) are presented below.

The final equation in terms of significant coded factors for ethanol response:

(2) $$\begin{array}{c} Y_{1}\left(\text{ethanol}\right)=-15.20+0.80X_{1}+1.40X_{2}+5.19X_{3}-0.02X_{1}X_{2}-0.03X_{1}X_{3}+0.26X_{2}X_{3}-0.01X_{1}^{2}-0.10X_{2}^{2}-5.05X_{3}^{2}. \end{array}$$

The final equation in terms of significant coded factors for fermentation efficiency response:

(3) $$\begin{array}{c} Y_{2}\left(\text{fermentation efficiency}\right)=-265.40+9.87X_{1}+44.90X_{2}+185.18X_{3}-0.97X_{1}X_{3}+3.95X_{2}X_{3}-0.06X_{12}-2.16X_{2}^{2}185.78X_{3}^{2}. \end{array}$$

*X*_1_, *X*_2_, and *X*_3_ are the code value of the tested variables: temperature, fermentation time, and cell ratio.

### 3.4. Effect of Temperature and Fermentation Time on Ethanol Production

**Figure 2:**
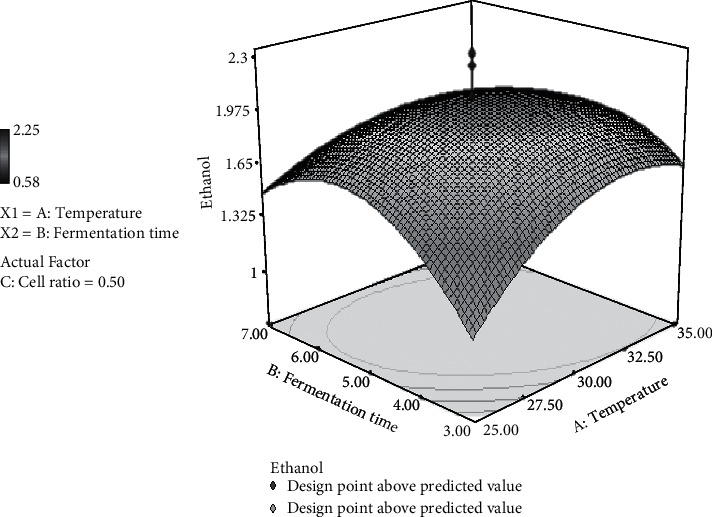
3D surface plot representing the interaction between temperature and fermentation time for the response ethanol by *S. cerevisiae/P. stipitis* in the simultaneous saccharification and cofermentation (SSCF) process.

Figure 2 shows the quadratic interaction between temperature and fermentation time for the ethanol response and indicates that temperature up to 32.5°C and fermentation time up to 5 days result in the production of high ethanol levels.

Temperature higher than 32.5°C and lower than 25°C leads to low ethanol production. This result is in accordance with Lin et al. [42] that found the optimum temperature of *S. cerevisiae* was 30–40°C for ethanol production. High temperatures approaching 40°C can interfere with transport activity in cells resulting in the production of ethanol decrease. In contrast, *S. cerevisiae* growth rate will decrease under 25°C due to low cell tolerance to ethanol [42]. Figure 2 also describes a quadratic relationship between the fermentation time toward ethanol production. The optimum ethanol production could be achieved for 5-day fermentation and decreased after 7-day fermentation. The decrease in ethanol content, along with the increase in fermentation time, is thought to be because ethanol is used by yeast as a carbon source [42].

### 3.5. Effect of Temperature and Cell Ratio on Ethanol Production

**Figure 3:**
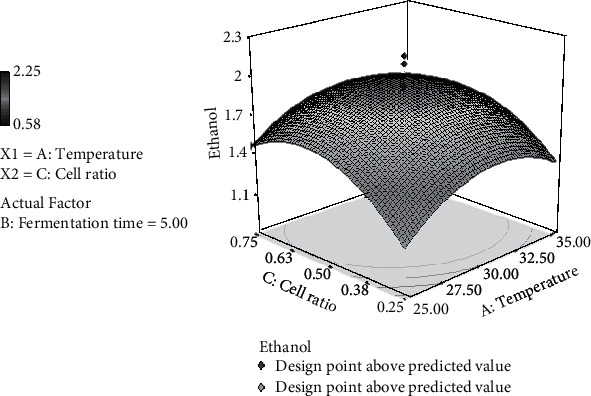
3D surface plot representing the interaction between temperature and cell ratio for the response ethanol by *S. cerevisiae/P. stipitis* in the simultaneous saccharification and cofermentation (SSCF) process.

A quadratic relationship was found between the cell ratio and ethanol production of *S. cerevisiae/P. stipitis* in the simultaneous saccharification and cofermentation (SSCF) process. The high ethanol production (1.66%, v/v) was achieved at cell ratio *S. cerevisiae/P. stipitis* of 0.50, and temperature of 30°C, for 3-day fermentation (Figure 3).

The cell ratio at a certain temperature significantly affected the amount of carbon source consumed by *S. cerevisiae and P. stipitis*, so it can affect the amount of ethanol content [39]. When cell ratio is 0.50 : 0.50 at 30°C for 5-day fermentation, *S. cerevisiae* and *Pichia stipitis* together actively utilize xylose and glucose as shown in Table 8. Xylose content decreased from 0.626% (v/v) to 0% and glucose content decreased from 6.163% to 0.0027%. When the cell ratio of *S. cerevisiae* was 0.75 at 35°C and 7-day fermentation, ethanol concentration reached 1.34% (v/v). In this condition, xylose content decreased from 1.062% (v/v) to 0.694% (v/v), suggesting that a higher consumption rate of glucose was performed by *S. cerevisiae* whereas *P. stipitis* could not consume the xylose maximally.

### 3.6. Effect of Fermentation Time and Cell Ratio on Ethanol Production

A quadratic relationship between fermentation time and cell ratio was observed on ethanol production.

**Figure 4:**
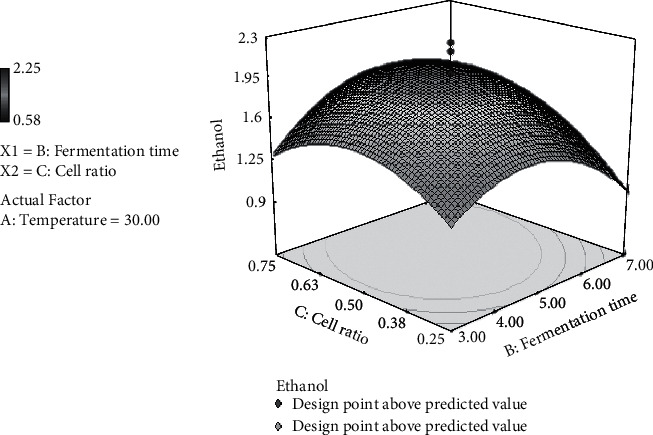
3D surface plot representing the interaction between cell ratio and fermentation time for the response ethanol by *S. cerevisiae/P. stipitis* in the simultaneous saccharification and cofermentation (SSCF) process.

Figure 4 shows that ethanol increased by the increase in the cell ratio and then decreased after reaching maximum ethanol content. The same phenomenon was also observed for fermentation time toward ethanol production [15, 16].

### 3.7. Effect of Temperature and Fermentation Time on Fermentation Efficiency

Fermentation efficiency can reach about 60% at a cell ratio of 0.5, 35°C, and 3-day fermentation. This result is in accordance with the data shown in Table 1 that shows the fermentation efficiency of about 62.63% under the cell ratio of *S. cerevisiae/P. stipitis* at 0.75 : 0.25, 35°C, and 3-day fermentation. *S. cerevisiae* is more active in using glucose than *P. stipitis* at 35°C since glucose consumption occurred during the cofermentation process but not for xylose (Table 8).

**Figure 5:**
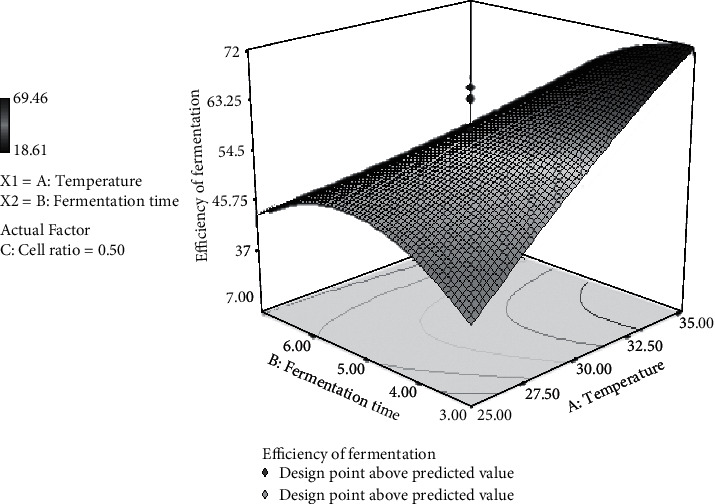
3D surface plot representing the interaction between temperature and fermentation time for the response fermentation efficiency by *S. cerevisiae/P. stipitis* in simultaneous saccharification and cofermentation (SSCF) process.

However, under the same cell ratio and temperature, the fermentation efficiency decreased to 45% when longer incubation (7 days) was performed (Figure 5).

The longer the fermentation time is, the lower the ethanol can be produced because ethanol will be consumed by *S. cerevisiae* as a carbon source to generate energy. On the contrary, a longer period of fermentation time will have a toxic effect on the growth of the microorganisms, especially in the batch mode due to the higher concentration of ethanol produced from the system [43, 44]. This relationship can be explained by analysis of variance (data not shown), which shows that there is a correlation between temperature and fermentation time on the efficiency of fermentation. The value of the correlation coefficient is −0.97. The negative values reflect an inverse relationship, meaning that the higher the temperature and the shorter the fermentation time, the more the fermentation efficiency. The result can be explained by the data shown in Table 8. When the temperature is 35°C, 3-day fermentation, and cell ratio of 0.75 : 0.25, the fermentation efficiency reaches 62.63%, but when the temperature is 25°C and 7-day fermentation, with the same cell ratios, the fermentation efficiency decreases to 40.16%.

### 3.8. The Effect of Temperature and Cell Ratio on Fermentation Efficiency

**Figure 6:**
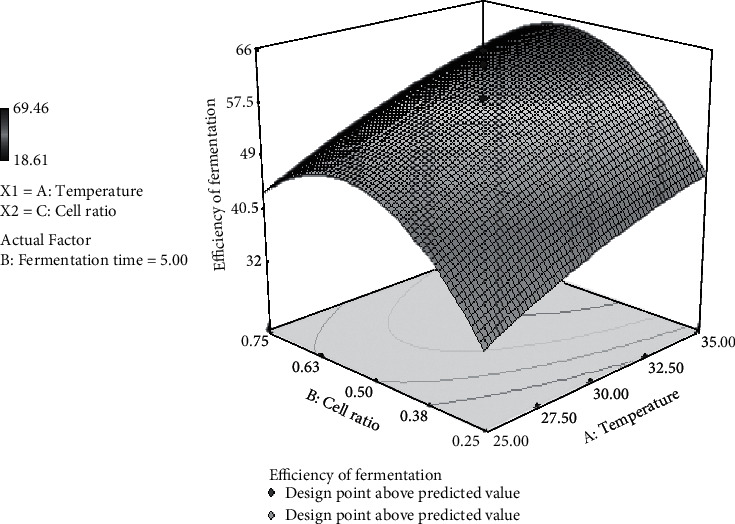
3D surface plot representing the interaction between cell ratio and temperature for the response fermentation efficiency by *S. cerevisiae/P. stipitis* in the simultaneous saccharification and cofermentation (SSCF) process.

Figure 6 shows the optimum fermentation efficiency in the range of 30°C, the cell ratio of 0.50 : 0.50, and 5-day fermentation. *S. cerevisiae* and *P. stipitis* can ferment together at a temperature and duration of fermentation. Results of analysis of variance show that there is no correlation between the ratio of cells with long fermentation on the efficiency of fermentation. However, the type of fermentation method is effected the fermentation efficiency, which fed-batch culture produced resulted in better cell concentration than batch culture did. In contrast, higher concentration of substrate was also found to affect the pH, viscosity, and the activity of the fermentation medium [45].

### 3.9. Effect of Fermentation Time and Cell Ratio on Fermentation Efficiency

**Figure 7:**
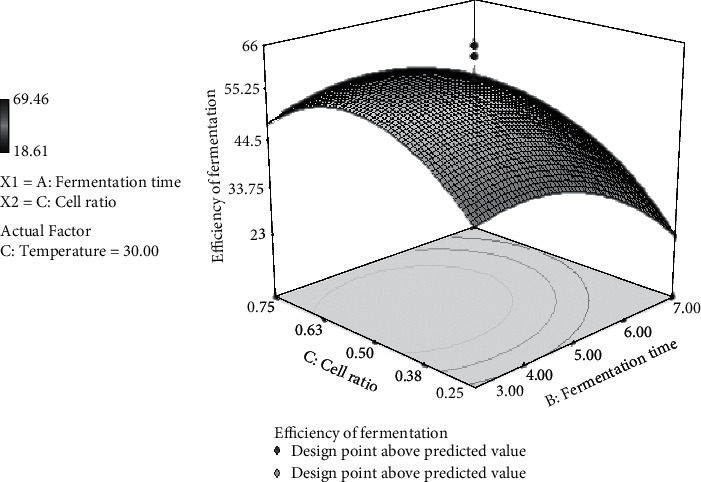
3D surface plot representing the interaction between cell ratio and fermentation time for the response fermentation efficiency by *S. cerevisiae/P. stipitis* in the simultaneous saccharification and cofermentation (SSCF) process.

Figure 7 shows that the cell ratio of 0.25 : 0.75, 3-day fermentation, and under 30°C achieved fermentation efficiency of about 41.5%. When the cell ratio is 0.50 : 0.50, fermentation efficiency increases to 54% and decreases to 50% when the cell ratio is 0.75 : 0.25. These data illustrate the quadratic relationship between the cell ratio and the efficiency of fermentation. The quadratic relationship is also found between fermentation time and fermentation efficiency. Fermentation efficiency reached 39%, 3-day fermentation, 30°C, cell ratio of 0.25 : 0.75; increased to 47% for 5-day fermentation; and decreased to approximately 25% for 7-day fermentation.

### 3.10. Validation for Optimal Simultaneous Saccharification and Cofermentation of Ethanol

Optimization of saccharification and cofermentation simultaneously (SSCF) temperature treatment, fermentation time, and cell ratio were statistically analyzed using Design-Expert software, version 7. The model predicted the optimal conditions as follows: cell ratio of *S. cerevisiae/P. stipitis* at 0.54; temperature at 33.45°C; and fermentation time at 4.22 days. The maximum yield prediction on ethanol and fermentation efficiency under optimal conditions is shown in Table 9.

The optimal condition at temperature of 33.45°C, fermentation time of 4.22 days, and cell ratio of *S. cerevisiae* at 0.54 are chosen by the program based on the levels of ethanol fermentation and highest efficiency. Validation is done by comparing the response of the actual experimental results with the predicted value of the program. The suitability of variables at the optimal point is tested and repeated three times based on temperature variables 33.45°C, 4.22 days, and cell ratio of *S. cerevisiae* at 0.54. Validation shows that the difference between actual experimental results and the predicted value of the program Design-Expert of ethanol content is 1.22% and 0.72% for fermentation efficiency (Table 10).

The differences between the experimental value and predicted value were found to be less than 5%, indicating the value of the independent variables' optimal point is quite suitable to produce an optimal response. Sun et al. [46] reported that the results of the experimental validation and predictive value of the program have an error rate of less than 5%, proving that the value of the optimum point variables has high suitability.

## 4. Conclusion

The SSCF process by *S. cerevisiae* and *P. stipitis* was systematically optimized using a design experiment. Alkaline peroxide combined with heat treatment successfully removed lignin until 93.22% of the treated oil palm trunk. Optimization of SSCF condition using the Doe model show that the coculture can work together to produce maximum ethanol and fermentation efficiency at 33, 45°C, 4.22 days, and cell ratio of 0.54 : 0.46 (*S. cerevisiae* NCYC 479 and *P. stipitis* NCYC 1541). This study could provide a strategy for the improvement of efficient ethanol production in the SSCF process of the oil palm trunk.

## Figures and Tables

**Table 1:** Optimization experiment design and ethanol production performance of *S. cerevisiae* and *P. stipitis* coculture by simultaneous saccharification and cofermentation (SSCF).

No.	Temperature (°C)	Fermentation time (days)	Cell ratio	Variable			Ethanol (% v/v) (*Y*_1_)	Fermentation efficiency (%) (*Y*_2_)
				*X*_1_	*X*_2_	*X*_3_		
1	35	7	0.75	+1	+1	+1	1.34	30.03
2	35	7	0.25	+1	+1	-1	0.87	18.61
3	35	3	0.75	+1	−1	+1	1.50	62.63
4	35	3	0.25	+1	−1	−1	1.45	49.21
5	25	7	0.75	−1	+1	+1	1.34	40.16
6	25	7	0.25	−1	+1	−1	0.64	19.18
7	25	3	0.75	−1	−1	+1	0.67	24.17
8	25	3	0.25	−1	−1	−1	0,58	20.99
9	38	5	0.50	+1.682	0	0	0.88	69.46
10	22	5	0.50	−1.682	0	0	1.34	46.50
11	30	8 days, 10 h	0.50	0	+1.682	0	0.78	22.41
12	30	1 day, 15 h	0.50	0	−1.682	0	0.82	53.43
13	30	5	0.92	0	0	+1.682	1.25	36.42
14	30	5	0.08	0	0	−1.682	0.78	22.53
15	30	5	0.50	0	0	0	2.18	63.20
16	30	5	0.50	0	0	0	1.77	51.32
17	30	5	0.50	0	0	0	1.84	53.36
18	30	5	0.50	0	0	0	2.25	65.24
19	30	5	0.50	0	0	0	1.89	55.20
20	30	5	0.50	0	0	0	2.00	57.99

**Table 2:** Chemical composition analysis of raw material after 30 mesh sieving.

Compound	Weight (g)	Lignin (% b/b)	Cellulose (% b/b)	Hemicellulose (% b/b)	Starch (% b/b)
≥595 *µ*m	36	19.40	33.90	14.30	25.18
≤595 *µ*m	64	18.58	30.21	14.52	35.00

**Table 3:** Chemical composition analysis of raw material after 80 mesh sieving.

Compound	Weight (g)	Lignin (% b/b)	Cellulose (% b/b)	Hemicellulose (% b/b)	Starch (% b/b)
≥177*µ*m	26	22.49	41.13	14.20	20.19
≤177um	74	19.06	28.19	14.70	36.84

**Table 4:** Chemical composition analysis of raw material after enzymatic hydrolysis.

Treatment	Weight (g)	Lignin (% b/b)	Cellulose (% b/b)	Hemicellulose (% b/b)	Starch (% b/b)
Before hydrolysis	100	22.49	41.13	14.20	20.19
After hydrolysis	72,27	17.73	51.45	16.63	12.24

**Table 5:** The lignin content before and after delignification.

Treatment	Lignin (% w/w)
1. Control	17.73
2. Alkali and heat treatment	18.76
3. Microwave-alkali treatment	18.23
4. Alkaline peroxide and heat treatment	3.39

**Table 6:** Chemical composition of raw materials after alkaline peroxide and heat treatment.

Treatment	Weight (g)	Lignin (% b/b)	Cellulose (% b/b)	Hemicellulose (% b/b)	Starch (% b/b)
Before	100	17.73	51.45	16.63	12.24
After	40	3.39	72.42	10.41	12.21

**Table 7:** Effect of alkaline peroxide and heat treatment on reducing sugar.

Treatment	Lignin (% w/w)	Reducing sugar^*∗*^ (% w/v)	Increasing of reducing sugar (%)
Before delignification	17.73	0.36	93.22
After delignification	3.39	5.31	

^*∗*^Hydrolysis of 5% (b/v) substrate, with 10 ml working volume using cellulose (Cellic Ctec2) 31.65 FPU/ml enzyme, pH buffer 5, at 50°C for 72 h.

**Table 8:** Xylose and glucose content of *S. cerevisiae/P. stipitis* simultaneous saccharification and cofermentation (SSCF) process.

No.	Temp (°C)	Fermentation (day)	Cell ratio	Simultaneous saccharification and cofermentation						Δ substrate	Ethanol (%)	Fermentation efficiency (%)
				Before			After					
				Xylose	Glucose	Total	Xylose	Glucose	Total			
1	35	7	0.75 : 0.25	1.062	8.435	9.497	0.694	0.054	0.748	8.749	1.34	30,03
2	35	7	0.25 : 0.75	1.062	8.435	9.497	0.295	0.037	0.332	9.165	0.87	18,61
3	35	3	0.75 : 0.25	0.759	7.002	7.761	0.970	2.095	3.065	4.696	1.50	62,63
4	35	3	0.25 : 0.75	0.759	7.002	7.761	0.915	1.068	1.983	5.778	1.45	49,21
5	25	7	0.75 : 0.25	0.841	5.702	6.543	0.000	0.000	0.000	6.543	1.34	40,16
6	25	7	0.25 : 0.75	0.841	5.702	6.543	0.000	0.000	0.000	6.543	0.64	19.18
7	25	3	0.75 : 0.25	0.419	5.042	5.461	0.000	0.026	0.026	5.435	0.67	24,17
8	25	3	0.25 : 0.75	0.419	5.042	5.461	0.000	0.042	0.042	5.419	0.58	20,99
9	38	5	0.50 : 0.50	1.124	10.319	11.443	1.201	7.758	8.959	2.484	0.88	69,46
10	22	5	0.50 : 0.50	0.780	4.920	5.700	0.000	0.050	0.050	5.650	1.34	46,50
11	30	8 days, 10 h	0.50 : 0.50	0.691	6.160	6.851	0.000	0.0027	0.027	6.824	0.78	22,41
12	30	1 day, 15 h	0.50 : 0.50	0.340	4.358	4.698	0.660	1.029	1.689	3.009	0.82	53,43
13	30	5	0.92 : 0.08	0.626	6.163	6.789	0.000	0.060	0.060	6.729	1.25	36,42
14	30	5	0.08 : 0.92	0.626	6.163	6.789	0.000	0.000	0.000	6.789	0.78	22,53
15	30	5	0.50 : 0.50	0.626	6.163	6.789	0.000	0.026	0.026	6.763	2.18	63,20
16	30	5	0.50 : 0.50	0.626	6.163	6.789	0.000	0.027	0.027	6.762	1.77	51,32
17	30	5	0.50 : 0.50	0.626	6.163	6.789	0.000	0.028	0.028	6.761	1.84	53,36
18	30	5	0.50 : 0.50	0.626	6.163	6.789	0.000	0.027	0.027	6.762	2.25	65,24
19	30	5	0.50 : 0.50	0.626	6.163	6.789	0.000	0.075	0.075	6.714	1.89	55,20
20	30	5	0.50 : 0.50	0.626	6.163	6.789	0.000	0.026	0.026	6.763	2.00	57,99

**Table 9:** Optimization predicted by Design-Expert program.

Response	Optimum point of SSCF under optimal conditions (33, 45°C, 4,22 days and cell ratio of *S. cerevisiae* 0,54)
Ethanol (%, v/v)	1,905
Fermentation efficiency	66,628

**Table 10:** Experimental validation.

Variables of SSCF			Ethanol (%)			Fermentation efficiency (%)		
Temperature (°C)	Fermentation time (days)	Cell ratio	Experiment	Predicted	% Difference	Experiment	Predicted	% Difference
33,45	4,22	0,54	1,87	1,91	2,09	65,91	66,62	1,07
33,45	4,22	0,54	1,90	1,91	0,52	66,14	66,62	0,72
33,45	4,22	0,54	1,89	1,91	1,05	66,37	66,62	0,38
Average			1,89	1,91	1,22	66,14	66,62	0,72

## Data Availability

The data used to support the findings of this study are included within the article.
